# ABO Blood Groups and Clinical Outcomes in Crimean–Congo Hemorrhagic Fever: Insights From a Large Turkish Cohort

**DOI:** 10.1155/jotm/3461989

**Published:** 2026-08-31

**Authors:** Caner Öksüz, Seyit Ali Büyüktuna, Murtaza Öz, Ertuğrul Keskin, Fatih Çubuk, Yasemin Çakır Kıymaz, Nazif Elaldı

**Affiliations:** ^1^ Department of Infectious Diseases and Clinical Microbiology, Faculty of Medicine, Sivas Cumhuriyet University, Sivas, Türkiye, cumhuriyet.edu.tr; ^2^ Department of Infectious Diseases and Clinical Microbiology, Sivas Numune Hospital, Sivas, Türkiye, saglik.gov.tr; ^3^ Department of Medical Microbiology, Sivas State Hospital, Sivas, Türkiye

**Keywords:** ABO, blood groups, CCHF, mortality, prognosis, Rh

## Abstract

Crimean–Congo hemorrhagic fever (CCHF) is a tick‐borne viral disease in which host‐related factors contributing to disease susceptibility and clinical outcomes remain incompletely understood. We aimed to evaluate the impact of ABO and Rh blood groups on susceptibility and prognosis in patients with CCHF and to investigate their relationship with epidemiological characteristics in a large real‐world cohort. This retrospective study included 1143 adult patients with PCR‐confirmed CCHF who were followed at our center between April 2015 and September 2024. ABO and Rh blood groups were determined using the Hitachi ElecSys 2010 Rack system, and hematological, biochemical, and coagulation parameters were evaluated simultaneously with CCHF PCR testing at hospital admission. Clinical outcomes, viral load, epidemiological characteristics, disease severity according to the Severity Grading Score (SGS), and laboratory findings were compared among blood groups. In addition, multivariable logistic regression analysis was performed to identify independent predictors of in‐hospital mortality. Of the 1143 patients, 732 (64.0%) were male and 411 (36.0%) were female; 548 patients had blood group A, 353 group O, 154 group B, and 88 group AB. Overall in‐hospital mortality was 8.5% (97/1143). No significant differences were observed among ABO or Rh blood groups regarding in‐hospital mortality, intensive care unit (ICU) requirement, viral load, or reported tick exposure. Although no deaths or ICU admissions occurred in the AB Rh‐negative subgroup, this subgroup comprised only 13 patients. Significant differences were identified only in amylase and lipase levels among blood groups, whereas SGS, ferritin, interleukin‐6, and other laboratory parameters were comparable. In the multivariate analysis, ABO and Rh blood groups were also not independently associated with in‐hospital mortality. These findings suggest that ABO and Rh blood groups are not associated with clinically meaningful differences in disease severity or prognosis in patients with CCHF. Further multicenter prospective studies are warranted to validate these findings.

## 1. Introduction

Crimean–Congo hemorrhagic fever (CCHF) is a severe tick‐borne viral disease caused by Crimean–Congo hemorrhagic fever virus (CCHFV) and remains an important public health problem in endemic regions, including Türkiye. The clinical spectrum of CCHF ranges from a mild self‐limiting febrile illness to severe disease characterized by hemorrhage, multiorgan failure, and death [1]. Although viral factors contribute to disease severity, increasing evidence suggests that host‐related factors also play an important role in determining susceptibility and clinical outcomes [2]. However, the host determinants influencing disease severity and prognosis remain incompletely understood.

The ABO blood group system has been associated with susceptibility and clinical outcomes in a variety of infectious diseases [3]. These associations are thought to result from the influence of ABO antigens on host–pathogen interactions, immune responses, endothelial function, and coagulation pathways [4]. Beyond their role in transfusion medicine, ABO antigens are expressed on vascular endothelial and epithelial cells, where they may modulate inflammatory responses and vascular homeostasis. In addition, ABO blood groups are associated with circulating levels of von Willebrand factor and factor VIII, providing a biologically plausible basis for investigating their potential role in diseases characterized by endothelial dysfunction and coagulopathy, such as CCHF [1, 4]. Previous studies have reported associations between ABO blood groups and susceptibility or disease severity in several viral, bacterial, and parasitic infections, although these relationships are not consistent across different pathogens [5, 6]. In contrast, evidence regarding the role of ABO/Rh blood groups in CCHF remains limited. Furthermore, to the best of our knowledge, no previous study has evaluated whether ABO/Rh blood groups are independently associated with in‐hospital mortality after adjustment for established prognostic factors in patients with CCHF.

Accordingly, the present study aimed to investigate the association of ABO and Rh blood groups with clinical outcomes, laboratory findings, disease severity, and epidemiological characteristics in a large cohort of adult patients with PCR‐confirmed CCHF. In addition, multivariable logistic regression analysis was performed to determine whether ABO/Rh blood groups were independently associated with in‐hospital mortality after adjustment for established prognostic factors.

## 2. Materials and Methods

The study included 1143 adult patients over 18 years of age diagnosed with CCHF and followed at our hospital between April 2015 and September 2024. Data were retrospectively obtained from patient files and analyzed.

For CCHF diagnosis, serum samples collected at hospital admission were tested using the RealStar CCHFV RT‐PCR kit (Altona, Germany) according to the manufacturer’s instructions. Analysis was performed on the Rotor‐Gene Q instrument (Qiagen, Germany). This kit allows for qualitative detection of CCHFV‐specific RNA in serum samples using RT‐PCR. Only adult patients (≥ 18 years) with a positive CCHF PCR result and followed for CCHF were included.

ABO/Rh blood groups, complete blood count, routine biochemical parameters (including liver enzymes, renal function tests, and pancreatic enzymes [amylase and lipase]), coagulation tests, and CCHF PCR were evaluated simultaneously as part of the routine clinical and laboratory assessment at hospital admission. ABO and Rh blood groups were determined using the Hitachi ElecSys 2010 Rack system.

Age, sex, history of tick attachment, length of hospital stay, intensive care unit (ICU) requirement, in‐hospital mortality, and clinical findings developing during hospitalization were analyzed in relation to blood groups. Thus, epidemiological data in CCHF patients were examined to highlight previously overlooked aspects of blood groups in infectious diseases. Mortality was defined as all‐cause in‐hospital mortality occurring during the index hospitalization.

Disease severity was scored using the Severity Grading Score (SGS) system defined by Bakır et al. [7]. The parameters and scoring system are shown in Table 1.

**TABLE 1:** Severity Grading Score (SGS) system.

Items	Classification	SGS points
Aspartate transaminase	< 5 X ULNV	0
	≥ 5 X ULNV	1

Alanine transaminase	< ULNV	0
	≥ ULNV	1

Lactate dehydrogenase	< 3 X ULNV	0
	≥ 3 X ULNV	1

White blood cells	< 10,000 cells/μL	0
	≥ 10,000 cells/μL	1

Hepatomegaly	No	0
	Yes	1

Organ failure	No	0
	Yes	1

Bleeding	No	0
	Yes	1

Age	< 60 years	0
	≥ 60 years	1

Platelets	≥ 100,000 cells/μL	0
	≥ 50,000, < 100,000 cells/μL	1
	< 50,000 cells/μL	2

Prolongation of PT	< 3 s	0
	≥ 3, < 6 s	1
	≥ 6 s	2

aPTT	< 70	0
	≥ 70	1

INR	< 1.6	0
	≥ 1.6	1

*Note:* s, seconds.

Abbreviations: aPTT, activated partial thromboplastin time; INR, international normalized ratio; PT, prothrombin time; ULNV, upper limit of normal value.

### 2.1. Statistical Analysis

Data were analyzed using IBM SPSS Statistics for Windows, Version 23.0 (IBM Corp., Armonk, NY, USA). Categorical variables were evaluated using the chi‐square test. The distribution of continuous variables was assessed with the Shapiro–Wilk test; non‐normally distributed data were compared using the Mann–Whitney U and Kruskal–Wallis tests. Continuous variables are presented as median (25th–75th percentile), and categorical variables as *n* (%). To identify independent predictors of in‐hospital mortality, a multivariable logistic regression analysis was performed. Variables were selected based on clinical relevance, previous evidence regarding prognostic factors in CCHF, and the number of mortality events in the cohort. Considering the presence of 97 mortality events, the number of variables entered into the model was limited to avoid overfitting, in line with the approximate rule of 10 events per variable. The model included ABO blood group, Rh factor, age, sex, alanine aminotransferase (ALT), aspartate aminotransferase (AST), platelet count, lactate dehydrogenase (LDH), and bleeding status. The SGS was not included in the model because it is a composite score derived from clinical and laboratory parameters, including age, platelet count, LDH, and bleeding. Including SGS together with its individual components could introduce multicollinearity and impair the interpretation of the independent effects of these variables. Odds ratios (ORs) with 95% confidence intervals (CIs) were calculated, and a two‐sided *p* value < 0.05 was considered statistically significant.

### 2.2. Ethical Approval

This study was conducted in accordance with the principles of the Declaration of Helsinki and was approved by the institutional ethics committee (Date: November 20, 2025; Decision No.: 2025‐11/08). Patient informed consent was not required because of the retrospective nature of the study and the use of anonymized clinical data, in accordance with the decision of the institutional ethics committee.

## 3. Results

A total of 1143 patients were included in the study. Of these, 732 (64%) were male and 411 (36%) were female. Blood group distribution revealed 548 patients with group A, 353 with group O, 154 with group B, and 88 with group AB (Table 2).

**TABLE 2:** Distribution of blood groups in patients with Crimean–Congo hemorrhagic fever.

Blood group	Rh+ (*n*, %)	Rh− (*n*, %)	Total (*n*, %)
O	300 (26.2%)	53 (4.6%)	353 (30.9%)
A	473 (41.4%)	75 (6.6%)	548 (47.9%)
B	139 (12.2%)	15 (1.3%)	154 (13.5%)
AB	75 (6.6%)	13 (1.1%)	88 (7.7%)
Total	987 (86.3%)	156 (13.6%)	1143 (100%)

The median age of patients by blood group was 50 (35–62) for group O, 53 (38–64) for group A, 49 (34–60) for group AB, and 55.5 (39–65) for group B. Overall, in‐hospital mortality occurred in 97 of the 1143 patients (8.5%).

When patients were grouped as A (Rh+ and Rh−), B (Rh+ and Rh−), O (Rh+ and Rh−), and AB (Rh+ and Rh−), no significant differences were observed regarding reported tick exposure, viral load, ICU requirement, or in‐hospital mortality (Table 3).

**TABLE 3:** Evaluation of in‐hospital mortality, epidemiological data, and viral load differences in relation to blood groups A (Rh+ and Rh−), B (Rh+ and Rh−), O (Rh+ and Rh−), and AB (Rh+ and Rh−).

Variable	A	B	AB	O	*p*
In‐hospital mortality	49 (9%)	14 (9%)	4 (5%)	30 (8%)	0.577
Intensive care unit (ICU) requirement	58 (11%)	17 (11%)	3 (3%)	32 (9%)	0.174
Reported tick exposure	358 (65%)	99 (64%)	62 (70%)	232 (66%)	0.734
Viral load (10^5^)	8.81 (1.13–64.5)	26.2 (1.4–104)	21 (0.9–140)	9.78 (0.7–42.7)	0.708

When patients were grouped by the Rh factor (Rh+ vs. Rh−), no significant differences were found in tick exposure, viral load, ICU requirement, or in‐hospital mortality (Table 4).

**TABLE 4:** Evaluation of in‐hospital mortality, epidemiological data, and viral load differences in relation to Rh+ and Rh− blood groups.

Variable	Rh−	Rh+	*p*
In‐hospital mortality	13 (8%)	84 (8%)	0.941
Intensive care unit (ICU) requirement	13 (8%)	97 (10%)	0.556
Reported tick exposure	102 (65%)	649 (65%)	0.869
Viral load (10^5^)	14.2 (1.9–86.8)	9.98 (0.8–63.7)	0.293

When patients were divided into eight groups (A Rh+, A Rh−, B Rh+, B Rh−, O Rh+, O Rh−, AB Rh+, and AB Rh−), epidemiological data, routine laboratory parameters, ferritin, interleukin‐6 (IL‐6), disease severity (SGS), and in‐hospital mortality were evaluated. Significant differences were observed only in age, amylase, and lipase values, while other parameters showed no statistically significant differences. Patients with A Rh− were the oldest, and AB Rh− were the youngest; this difference was statistically significant. The highest amylase values were detected in O Rh− patients, which was statistically significant. Statistically, the highest lipase values were observed in the B Rh + group (Table 5).

**TABLE 5:** Comparison of blood groups A Rh+, A Rh−, B Rh+, B Rh−, O Rh+, O Rh−, AB Rh+, and AB Rh− in terms of epidemiological data, routine hematological parameters, ferritin, interleukin‐6 (IL‐6), disease severity (SGS), and in‐hospital mortality.

Variable	A Rh− (75)	A Rh+ (473)	B Rh− (15)	B Rh+ (139)	AB Rh− (13)	AB Rh+ (75)	O Rh− (53)	O Rh+ (300)	*p*
Age	59 (46–66)	52 (37–63)	49 (44–69)	56 (39–65)	43 (32–57)	50 (34–61)	48 (36–59)	51 (34–63)	**0.032**
Sex	45 (60%)	297 (62%)	7 (46%)	84 (60%)	9 (69%)	47 (62%)	37 (69%)	206 (68%)	0.391
In‐hospital mortality	8 (10%)	41 (9%)	1 (7%)	13 (9%)	0 (0%)	4 (6%)	4 (7%)	26 (9%)	0.893
Intensive care unit (ICU) requirement	8 (10%)	50 (10%)	1 (7%)	16 (11%)	0 (0%)	3 (4%)	4 (7%)	28 (9%)	0.575
Reported tick exposure	51 (7%)	307 (6%)	11 (6%)	88 (6%)	9 (7%)	53 (7%)	31 (6%)	201 (7%)	0.850
Length of hospital stay	6 (4–8)	6 (4–8)	6 (4–8)	6 (5–8)	7 (4–8)	6 (5–8)	7 (4–8)	6 (4–8)	0.768
WBC	2.6 (2.2–3.8)	2.8 (2.0–4.3)	2.2 (1.4–2.7)	2.8 (1.9–3.9)	3.2 (2.4–4.0)	2.8 (2.1–4.4)	2.5 (1.8–3.3)	2.7 (1.8–4.0)	0.344
HB	14.1 (12.5–14.9)	14.1 (12.8–15.1)	13.4 (12.7–14.7)	14.4 (13.4–15.4)	15.0 (13.3–15.6)	14.3 (13.2–15.2)	14.4 (12.9–15.5)	14.3 (13.2–15.3)	0.071
PLT	83 (43–131)	93 (54–132)	76 (44–121)	94 (49–130)	120 (52–166)	96 (57–137)	79 (38–121)	92 (51–125)	0.393
PT	11.7 (10.6–13.2)	11.8 (10.3–13.6)	12.1 (10.9–14.2)	11.8 (10.8–13.7)	11.8 (10.7–13.6)	11.6 (10.3–13.7)	11.6 (10.1–13.9)	11.9 (10.5–13.6)	0.939
aPTT	31.1 (25.7–36.3)	30.3 (26.5–36.3)	31.6 (27.4–33.0)	30.2 (25.2–37.3)	29.7 (24.1–32.3)	29.7 (26.2–34.9)	30.9 (27.8–42.3)	30.8 (26.6–35.7)	0.603
D‐dimer	1520 (678–4840)	1480 (659–5870)	1460 (650–3190)	1560 (667–5975)	1325 (521–11015)	1620 (539–5070)	1380 (692–4805)	2086 (731–6107)	0.785
BUN	16.4 (12.3–22.5)	15.4 (11.3–21.2)	13.3 (11.8–18.8)	16.3 (11.6–20.9)	12.8 (11.4–15.8)	14.2 (10.7–18.4)	16.3 (10.9–20.5)	15.7 (11.5–20.2)	0.304
CRE	0.87 (0.73–1.17)	0.85 (0.72–1.06)	0.80 (0.69–0.98)	0.90 (0.74–1.08)	0.78 (0.65–0.83)	0.83 (0.72–0.96)	0.87 (0.72–1.05)	0.88 (0.72–1.07)	0.284
ALT	51 (27–93)	53 (27–114)	82 (16–159)	53 (29–107)	42 (21–98)	48 (23–105)	55 (25–133)	48 (25–92)	0.709
AST	92 (42–163)	95 (45–222)	150 (26–288)	88 (46–224)	86 (43–166)	72 (40–175)	75 (43–286)	87 (45–210)	0.938
LDH	377 (264–581)	382 (268–598)	417 (260–504)	397 (262–602)	346 (272–467)	325 (242–522)	359 (271–719)	404 (267–576)	0.693
Amylase	75 (53–104)	74 (56–106)	60 (39–81)	82 (61–125)	84 (71–113)	72 (51–89)	85 (67–126)	80 (60–108)	**0.005**
Lipase	51 (39–79)	52 (36–81)	68 (40–81)	72 (41–117)	48 (38–68)	50 (36–101)	60 (36–125)	55 (37–81)	**0.012**
Ferritin	1402 (222–6514)	1494 (480–8446)	1603 (199–8935)	2611 (689–12793)	1162 (628–12288)	1032 (363–13220)	2663 (550–6269)	1846 (527–7957)	0.698
IL‐6	21.3 (9.0–31.1)	20.5 (9.8–54.4)	22.1 (14.7–84.5)	16.9 (9.3–52.5)	14.2 (7.1–18.5)	16.0 (10.6–54.9)	19.9 (12.5–43.5)	20.4 (10.9–43.9)	0.919
SGS	2 (1–5)	2 (1–4)	3 (1–5)	3 (1–4)	1 (0–3)	2 (1–3)	2 (1–5)	2 (1–4)	0.236

*Note:* HB, hemoglobin; PLT, platelet; ALT, alanine transaminase; AST, aspartate transaminase; LDH, lactate dehydrogenase; IL‐6, interleukin‐6; CRE, creatinine. Bold values indicate statistical significance.

Abbreviations: aPTT, activated partial thromboplastin time; BUN, blood urea nitrogen; PT, prothrombin time; SGS, Severity Grading Score; WBC, white blood cell.

No significant differences were observed among blood groups regarding clinical findings during hospitalization (Table 6).

**TABLE 6:** Evaluation of patients’ clinical findings according to blood groups.

Variable	A Rh− (75)	A Rh+ (473)	B Rh− (15)	B Rh+ (139)	AB Rh− (13)	AB Rh+ (75)	O Rh− (53)	O Rh+ (300)	*p*
Presence of petechiae and ecchymosis	11 (14%)	82 (17%)	1 (6%)	27 (19%)	2 (1%)	8 (10%)	12 (22%)	46 (15%)	0.547
Active bleeding	12 (16%)	75 (15%)	1 (6%)	24 (17%)	1 (0.7%)	7 (9%)	13 (24%)	41 (13%)	0.316
Fever	61 (81%)	392 (82%)	14 (93%)	118 (84%)	12 (92%)	60 (80%)	48 (90%)	249 (83%)	0.660
Nausea and vomiting	39 (52%)	251 (53%)	8 (53%)	78 (56%)	7 (53%)	32 (42%)	31 (58%)	155 (51%)	0.713
Diarrhea	18 (24%)	144 (30%)	2 (13%)	43 (30%)	3 (24%)	15 (20%)	17 (32%)	96 (32%)	0.343

To determine whether ABO and Rh blood groups were independently associated with in‐hospital mortality, a multivariable logistic regression analysis was performed (Table 7). Increasing age (OR: 1.030, 95% CI: 1.014–1.046; *p* < 0.001) and higher LDH levels (OR: 1.001, 95% CI: 1.001–1.002; *p* < 0.001) were independently associated with increased in‐hospital mortality, whereas higher platelet counts were independently associated with lower in‐hospital mortality (OR: 0.993, 95% CI: 0.986–0.999; *p* = 0.025). In contrast, the ABO blood group, Rh factor, sex, ALT, AST, and bleeding status were not independently associated with in‐hospital mortality.

**TABLE 7:** Multivariable logistic regression analysis of factors associated with in‐hospital mortality in patients with Crimean–Congo hemorrhagic fever (CCHF).

Variable	Odds ratio (OR)	95% confidence interval	*p* value
ABO blood group			
B vs A	0.877	0.501–1.533	0.644
AB vs A	0.571	0.166–1.965	0.374
O vs A	0.948	0.434–2.071	0.893
Rh factor			
Rh‐negative vs Rh‐positive	0.738	0.350–1.556	0.425
Age (years)	1.030	1.014–1.046	**< 0.001**
Male sex (vs female)	0.633	0.369–1.085	0.096
ALT (U/L)	0.999	0.997–1.000	0.073
AST (U/L)	1.000	0.9996–1.001	0.655
Bleeding (yes vs no)	0.563	0.303–1.046	0.069
Platelet count (× 10^3^/μL)	0.993	0.986–0.999	**0.025**
LDH (U/L)	1.001	1.001–1.002	**< 0.001**

*Note:* ALT, alanine aminotransferase; AST, aspartate aminotransferase; PLT, platelet count; LDH, lactate dehydrogenase. Bold values indicate statistical significance.

Abbreviations: CI, confidence interval; OR, odds ratio.

## 4. Discussion

Our study included 1143 adult patients with positive CCHF PCR results followed at our hospital. Of these, 732 (64%) were male, and 411 (36%) female. Previous studies in Türkiye have also reported a predominance of male patients with CCHF [8–11], consistent with our findings.

The association between infectious diseases and ABO blood groups has been a longstanding topic of interest and has been investigated across multiple conditions. A meta‐analysis examining the relationship between ABO blood groups and tuberculosis identified the AB blood group as a significant risk factor for tuberculosis, particularly in Africa and India [12]. This finding underscores the importance of considering regional variations when assessing the link between blood groups and disease susceptibility.

In a study summarizing the literature on ABO blood groups and COVID‐19, blood group O was generally associated with lower rates of *SARS-CoV-2* infection, whereas blood group A was frequently reported as a risk factor. Although findings on the risk of severe disease were inconsistent, blood group A was most commonly linked to severe COVID‐19 and increased mortality [13]. Researchers have noted substantial heterogeneity in studies evaluating the effect of ABO blood groups on the risk and severity of *SARS-CoV-2* infection, likely due to differences in study design, the type and size of case and control groups, and participants’ ethnic backgrounds.

Similarly, a study investigating the association between ABO and Rh antigens and HBV infection among young Nigerian adults reported that the B Rh− blood group carried a higher risk of HBV acquisition [14], whereas a meta‐analysis of 38 studies indicated that individuals with blood group B had the lowest risk for HBV infection [15]. This discrepancy may be explained by differences in study populations and settings, with the meta‐analysis focusing on non‐African cohorts and blood donors or hospitalized patients, as opposed to young undergraduate students [14].

In a German blood donor cohort, ABO blood group was not identified as a significant risk factor for Parvovirus B19 infection; however, it was notable that all cases occurred in the Rh+ cohort [16]. Additionally, a study assessing the prevalence of *Toxoplasma gondii* found no significant association with ABO blood groups [17]. In South Africa, analysis of blood donors from eight of nine provinces demonstrated that ABO blood group polymorphisms did not influence susceptibility to HIV infection [18].

These findings suggest that ABO blood groups are not universally risk factors for infectious diseases. Blood group antigens may directly modulate infections by serving as receptors or co‐receptors for microorganisms and toxins, or indirectly via blood group antibodies elicited by bacteria or enveloped viruses expressing blood group‐like antigens [19]. For microorganisms where no association with blood groups has been observed, such direct or indirect interactions are likely absent.

Several biological mechanisms may explain why ABO blood groups have been investigated as potential determinants of susceptibility and clinical outcomes in infectious diseases. ABO antigens are expressed not only on erythrocytes but also on vascular endothelial and epithelial cells, where they may influence host–pathogen interactions and immune responses. Moreover, ABO blood groups are associated with circulating levels of von Willebrand factor and factor VIII, both of which play important roles in endothelial integrity, coagulation, and thromboinflammatory processes [20]. Since severe CCHF is characterized by endothelial injury, increased vascular permeability, activation of coagulation pathways, and an exaggerated inflammatory response, it is biologically plausible that ABO blood groups could influence disease severity and clinical outcomes [21, 22]. However, the absence of such an association in our cohort suggests that the contribution of the ABO and Rh blood group system, if present, is likely modest compared with other host and viral factors involved in CCHF pathogenesis.

The relationship between blood groups and mortality in CCHF has previously been assessed in smaller patient cohorts [23, 24]. Barkay and Karakeçili’s study included a total of 368 patients, of whom 7 (1.9%) died [23]. No significant differences in mortality rates were observed between blood groups in this study (*p* = 0.956) [23]. In the study population of Duygu and Kaya, which included 292 patients, 13 (4.45%) deaths were recorded [24]. When compared with the general population and the study cohort, a high proportion of deceased patients had the AB blood group. The prevalence of the AB Rh− blood group was reported as 1.03% and 2.9% in blood bank samples and in general CCHF patients, respectively, whereas in fatal CCHF cases this proportion reached 23.07%. This difference was statistically significant (*p* < 0.001) [24].

In our study, among 1143 patients, 97 (8.5%) experienced a fatal course. The higher in‐hospital mortality rate observed in our cohort may be explained by the larger patient population over a longer study period, as well as the fact that our hospital, being a regional referral center, receives severe cases transferred from other hospitals. Similar to Barkay and Karakeçili’s findings [23], no significant differences in in‐hospital mortality rates were observed between blood groups in our cohort. When patients were grouped as Rh+ and Rh−, no significant difference in in‐hospital mortality rates was detected. Similarly, when patients were categorized into two groups (Rh+ vs. Rh−), four groups (A, B, O, and AB), or eight groups according to the ABO/Rh system, no significant differences were observed in the need for ICU. Nevertheless, it is noteworthy that no in‐hospital mortality or ICU requirement was observed in the AB Rh− subgroup, which may be explained by the small size of this group (*n* = 13, 1.1%). Further studies are required to clarify this observation.

To further determine whether ABO and Rh blood groups were independently associated with mortality, we performed a multivariable logistic regression analysis adjusting for clinically relevant prognostic variables. After adjustment, neither the ABO blood group nor Rh factor remained independently associated with in‐hospital mortality. Instead, increasing age and higher LDH levels were independently associated with mortality, whereas higher platelet counts were associated with lower mortality. These findings indicate that established clinical and laboratory markers of disease severity have a substantially greater prognostic value than the ABO/Rh blood group system in patients with CCHF [7].

As viral load is a major determinant of mortality, blood groups were also assessed for differences in viral load [25]. No significant differences in viral load were observed when patients were stratified into four groups (A, B, O, and AB) or two groups (Rh+ vs. Rh−).

Blood groups were additionally evaluated in terms of routine laboratory parameters, ferritin, interleukin‐6 (IL‐6), and disease severity score (SGS). Significant differences were found in amylase and lipase values. The highest amylase levels were observed in O Rh− patients, and this difference was statistically significant. The B Rh+ group demonstrated the highest lipase levels, which were also statistically significant. The clinical significance of the observed differences in amylase and lipase levels among ABO and Rh blood groups remains uncertain. Pancreatic involvement has only rarely been reported in patients with CCHF, and its pathophysiological basis has not been fully elucidated [26]. Current evidence suggests that pancreatic enzyme elevation in CCHF is more likely to occur secondary to systemic disease rather than as a result of direct pancreatic tropism [1]. Severe CCHF is characterized by widespread endothelial injury, increased vascular permeability, dysregulated inflammatory responses, and coagulation abnormalities, all of which may contribute to microvascular ischemia and multiorgan involvement, including the pancreas [21, 22]. Nevertheless, elevated amylase and lipase levels do not necessarily indicate clinically significant pancreatitis and may reflect nonspecific enzyme release during severe systemic illness. In our study, although amylase and lipase levels differed significantly among ABO and Rh blood groups, these differences were not accompanied by corresponding differences in disease severity, ICU admission, or in‐hospital mortality. Therefore, the clinical relevance of these findings remains uncertain and should be interpreted cautiously until confirmed by larger studies incorporating clinical, radiological, and serial biochemical assessment of pancreatic involvement.

Furthermore, our study explored the relationship between blood groups and epidemiological data, an often‐overlooked aspect in the evaluation of infectious diseases. Previously, the blood group distribution of 50,441 individuals aged over 18 who presented to our hospital for various reasons between 2018 and 2020 was reported [27]. In that study, 19,387 individuals (38.4%) were A Rh+, 14,045 (27.8%) O Rh+, 7202 (14.2%) B Rh+, 3445 (6.8%) AB Rh+, 2781 (5.5%) A Rh−, 2060 (4.08%) O Rh−, 1027 (2.03%) B Rh−, and 500 (0.9%) AB Rh−. Reflecting the general blood group distribution of our region, the most common blood groups among CCHF patients were A Rh+, O Rh+, and B Rh+, whereas AB Rh− was the least frequent. These results indicate that the blood group distribution among CCHF patients is similar to that of the general population, suggesting no specific blood group preference by ticks in CCHF transmission. Additionally, no differences were observed between blood groups in patients’ reports of tick exposure. Furthermore, the assessment of tick exposure relied on patient self‐report rather than objective confirmation. Because tick bites may frequently go unnoticed or be forgotten, this variable is susceptible to recall and misclassification bias, potentially limiting the interpretation of analyses involving reported tick exposure.

ABO blood group distributions vary considerably among different geographical regions and ethnic populations [28]. Therefore, although our findings indicate that ABO/Rh blood groups are not independently associated with in‐hospital mortality in this large cohort of patients with CCHF from an endemic region of Türkiye, these results may not be directly generalizable to populations with different genetic backgrounds or blood group distributions. Future multicenter studies involving diverse geographical regions and populations are warranted to determine whether our findings are consistent across different epidemiological settings.

## 5. Conclusion

In conclusion, this large‐scale Turkish cohort of 1143 patients demonstrates that ABO and Rh blood groups are not independently associated with in‐hospital mortality in patients with CCHF after adjustment for established prognostic factors. Although statistically significant differences were observed in amylase and lipase levels among specific ABO/Rh subgroups, these findings were not associated with disease severity, ICU admission, or in‐hospital mortality and therefore should be interpreted with caution. Nevertheless, these subgroups may warrant closer monitoring of pancreatic enzyme levels during the course of the disease. Contrary to the limited and conflicting findings reported in previous studies, our results indicate that the ABO/Rh blood group system is unlikely to represent a major independent prognostic determinant in CCHF. Further multicenter prospective studies involving geographically and ethnically diverse populations are warranted to externally validate these findings, clarify the clinical significance of the observed pancreatic enzyme differences, and better define the prognostic implications for rarer subgroups, such as AB Rh−.

## Author Contributions

Caner Öksüz: conceptualization, study design, data curation, formal analysis, investigation, methodology, supervision, visualization, writing–original draft, and writing–review and editing. Seyit Ali Büyüktuna: data curation, formal analysis, investigation, methodology, and writing–review and editing. Murtaza Öz: data collection, formal analysis,resources, investigation, and writing–review and editing. Ertuğrul Keskin: data collection, clinical evaluation, resources, and writing–review and editing. Fatih Çubuk: laboratory analysis, data acquisition, validation, and writing–review and editing. Yasemin Çakır Kıymaz: methodology, data interpretation, supervision, and writing–review and editing. Nazif Elaldı: conceptualization, methodology, supervision, critical revision of the manuscript, and writing–review and editing.

## Funding

This research received no specific grant from any funding agency in the public, commercial, or not‐for‐profit sectors. All aspects of the study, including design, data collection, analysis, and manuscript preparation, were carried out without external financial support.

## Disclosure

All authors have read and approved the final version of the manuscript and agree to be accountable for all aspects of the work.

## Ethics Statement

This study was conducted in accordance with the principles of the Declaration of Helsinki and was approved by the institutional ethics committee (Date: November 20, 2025; Decision No.: 2025‐11/08). Patient informed consent was not required because of the retrospective nature of the study and the use of anonymized clinical data, in accordance with the decision of the institutional ethics committee.

## Consent

Please see the Ethics Statement.

## Conflicts of Interest

The authors declare no conflicts of interest.

## Data Availability

The datasets generated and/or analyzed during the current study are available from the corresponding author on reasonable request.
